# Pathologic collision of urinary bladder urothelial carcinoma with small cell carcinoma: a case report

**DOI:** 10.1186/s13000-023-01369-x

**Published:** 2023-07-11

**Authors:** Wei Jiang, Chi Pan, Wei Guo, Zhen Xu, Qingtao Ni, Yashi Ruan

**Affiliations:** 1 Department of Urological Surgery, The Affiliated Taizhou People’s Hospital of Nanjing Medical University, Taizhou School of Clinical Medicine, Nanjing Medical University, Taizhou, 225300 Jiangsu P.R. China; 2 Department of General Surgery, The Affiliated Taizhou People’s Hospital of Nanjing Medical University, Taizhou School of Clinical Medicine, Nanjing Medical University, Taizhou, 225300 Jiangsu P.R. China; 3 Department of Oncology, The Affiliated Taizhou People’s Hospital of Nanjing Medical University, Taizhou School of Clinical Medicine, Nanjing Medical University, Taizhou, 225300 Jiangsu P.R. China

**Keywords:** Case report, Pathologic collision, Small cell carcinoma, Urinary bladder cancer, Urothelial carcinoma

## Abstract

**Background:**

Urothelial carcinoma is a major subtype of bladder cancer and small cell carcinoma (SCC) is a rare type of cancer in clinical practice. Pathologic collision of urinary bladder urothelial carcinoma with SCC is not common in clinical settings.

**Case presentation:**

Here, we report a patient with high-grade papillary carcinoma which changed to collision tumor with SCC. The patient underwent radical cystectomy; however, neck and mediastinum lymph nodes metastases were detected 11 months after the operation. The lymph nodes were diagnosed pathologically as SCC. Chemoradiotherapy was subsequently prescribed. Unfortunately, this patient died of COVID-19 in early 2023.

**Discussion:**

We hypothesized the mechanism underlying this pathological transformation. For patients with urothelial bladder cancer, pathological analysis should be conducted to allow standardized and persistent treatment. Moreover, drugs should be selected depending on the type of pathology, especially for patients who develop relapse, since collision tumor or other pathological tumors may be present.

**Conclusions:**

We recommend that radical cystectomy be performed early enough for patients with non-muscle invasive bladder cancer, who are at a high risk of tumor recurrence. However, this conclusion needs to be validated in a larger number of patients.

## Key points

• Clinicians should be alert to alterations in pathological processes.
• Radical cystectomy maybe necessary for patients with recurrent bladder cancer.

## Background

Bladder cancer is the tenth most common cancer in the world, accounting for up to 3% of all new cancer diagnoses [1]. The incidence of bladder cancer varies with gender, with men having a 3 to 4 times higher morbidity than women [2]. Previous statistics indicate that about 420,000 cases of bladder cancer are diagnosed annually, among which 16,500 people die [3], and the in situ survival rate is about 96% [4]. Based on whether cancer cells can infiltrate the muscular layer of the bladder, bladder cancer is divided into two subtypes, namely non-muscle invasive bladder cancer (NMIBC) and muscular invasive bladder cancer (MIBC) [5]. NMIBC can be further divided into stage Ta, Tis and T1 stage bladder cancer, whereas MIBC is T2-4 stage bladder cancer [6]. Transurethral resection of bladder tumor (TURBT) is the primary treatment modality for NMIBC [7]. Bipolar-TURBT has a lower risk of overall complications compared with monopolar-TURBT in the treatment of NMIBC [8]. It has been reported that MIBC occurs in 20–25% of all bladder cancer cases [9], while about 10–15% of all MIBC patients are originally diagnosed with NMIBC, which later transforms to MIBC [10]. Although radical cystectomy and radio/chemotherapy are the major treatment modalities for MIBC, approximately one quarter of all MIBC patients show poor prognosis following treatment [3].

Urinary bladder carcinoma has two major subtypes, namely urothelial carcinoma, which accounts for > 90% of all cases, and non-urothelial carcinoma which includes squamous cell carcinoma, adenocarcinoma and small cell carcinoma (SCC), and accounts for < 1% of all urinary bladder cancer cases [11, 12]. In this report, we present a rare case of pathologic collision of urinary bladder urothelial carcinoma with squamous cell carcinoma (SCC) in a bladder cancer patient at our hospital.

## Case presentation

**Fig. 1:**
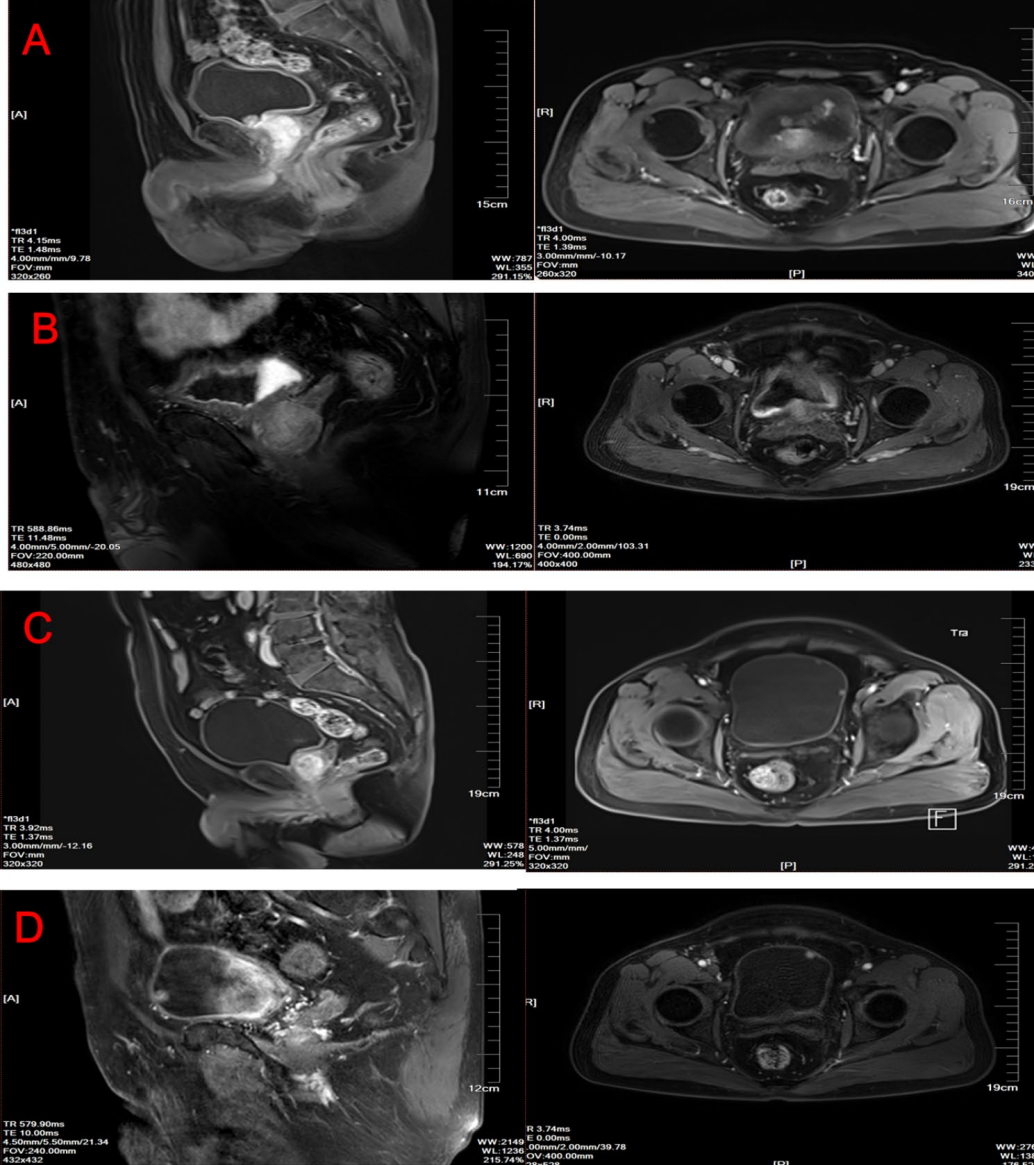
Magnetic resonance imaging of bladder cancer patient. **A)** MRI image revealed irregular and nodular enhancement, the largest which had a size of 2.9*1.2 cm in the bladder (sagittal and coronal) in November 2017. **B)** Pelvic MRI showing thickening of the posterior urinary bladder, and obvious intensification after enhancement (sagittal and coronal) in May, 2018. **C)** Pelvic MRI showing multiple nodular enhancement, the largest which had a size 0.6*0.8 cm in the bladder (sagittal and coronal) in November, 2018. **D)** Pelvic MRI showing signal abnormalities with the size of 0.9*0.7 cm in the bladder (sagittal and coronal) in June, 2019

**Fig. 2:**
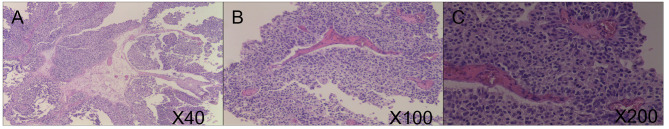
Pathological results showing that the tumor was high-grade papillary carcinoma. Hematoxylin and eosin (H&E)-stained sections at: **(A)** × 40; **(B)** × 100; **(C)** × 200

A 64-year-old man visited our hospital because of urinary urgency, frequent urination and dysuria in November 2017. He had 10- and 7-year histories of hypertension and cerebral infarction, respectively. Therefore, the patient was maintained on a long-term regimen of oral irbesartan, aspirin, and atorvastatin. He had no previous medical history or family history of tumors. Based on the clinical features, a diagnosis of prostatic hyperplasia was considered. Furthermore, prostate MRI revealed bladder tumor. To characterize the bladder tumor, pelvic magnetic resonance imaging (MRI) was conducted. Pelvic MRI revealed irregular and nodular enhancement in the bladder, with the largest enhancement measuring 2.9*1.2 cm (Fig. 1A). TURBT was performed to facilitate the diagnosis and as a therapeutic modality on 13th November 2017, with perfusion of “1 *g* gemcitabine” conducted during the surgical procedure. Pathological results revealed that the tumor was high-grade papillary carcinoma (G3) (Fig. 2), with a total size of 3.5*2.5*1.5 cm, and had infiltrated the lamina propria. Therefore, the patient was diagnosed as NMIBC with T1N0M0, according to the TNM classification by Union for International Cancer Control (UICC). Since high-risk T1G3 bladder cancer is normally characterized by poor prognosis and high recurrence rate [13], the patient was put on bladder infusion chemotherapy. Due to financial considerations, the patient opted against utilizing Bacillus Calmette Guerin (BCG). After a comprehensive decision-making process, gemcitabine was selected as an alternative treatment. Following a single administration of “gemcitabine 1* g*,“ the patient refused further bladder infusion due to the onset of bladder irritation. On 2nd February 2018, he was administered with a second treatment with TURBT, according to related literature [14]. Pathological findings revealed moderate-severe atypical hyperplasia.

The patient suffered from recurrent episodes of hematuria, and hence was admitted to our hospital on 3rd May, 2018. MRI scan of the pelvic revealed thickening of the posterior urinary bladder, and obvious intensification after enhancement (Fig. 1B). The condition was considered bladder cancer recurrence, for which radical cystectomy was recommended. However, the patient expressed concerns of poor overall quality of life following the operation and refused to undergo the surgical procedure. TURBT was again performed and pathology showed high-grade papillary carcinoma.

On 21st November, 2018, recurrent hematuria was detected. Pelvic MRI revealed multiple nodular enhancement, the largest of which was in the bladder with a size of 0.6*0.8 cm (Fig. 1C). Radical cystectomy was also recommended, but the patient declined. A repeat TURBT was, therefore, performed. On 26th June, 2019, re-emergence of hematuria was observed in the patient. Pelvic MRI revealed signal abnormalities, with a size of 0.9*0.7 cm, in the bladder (Fig. 1D). TURBT was performed again, with pathological results showing no significant changes compared to before operation.

**Fig. 3:**
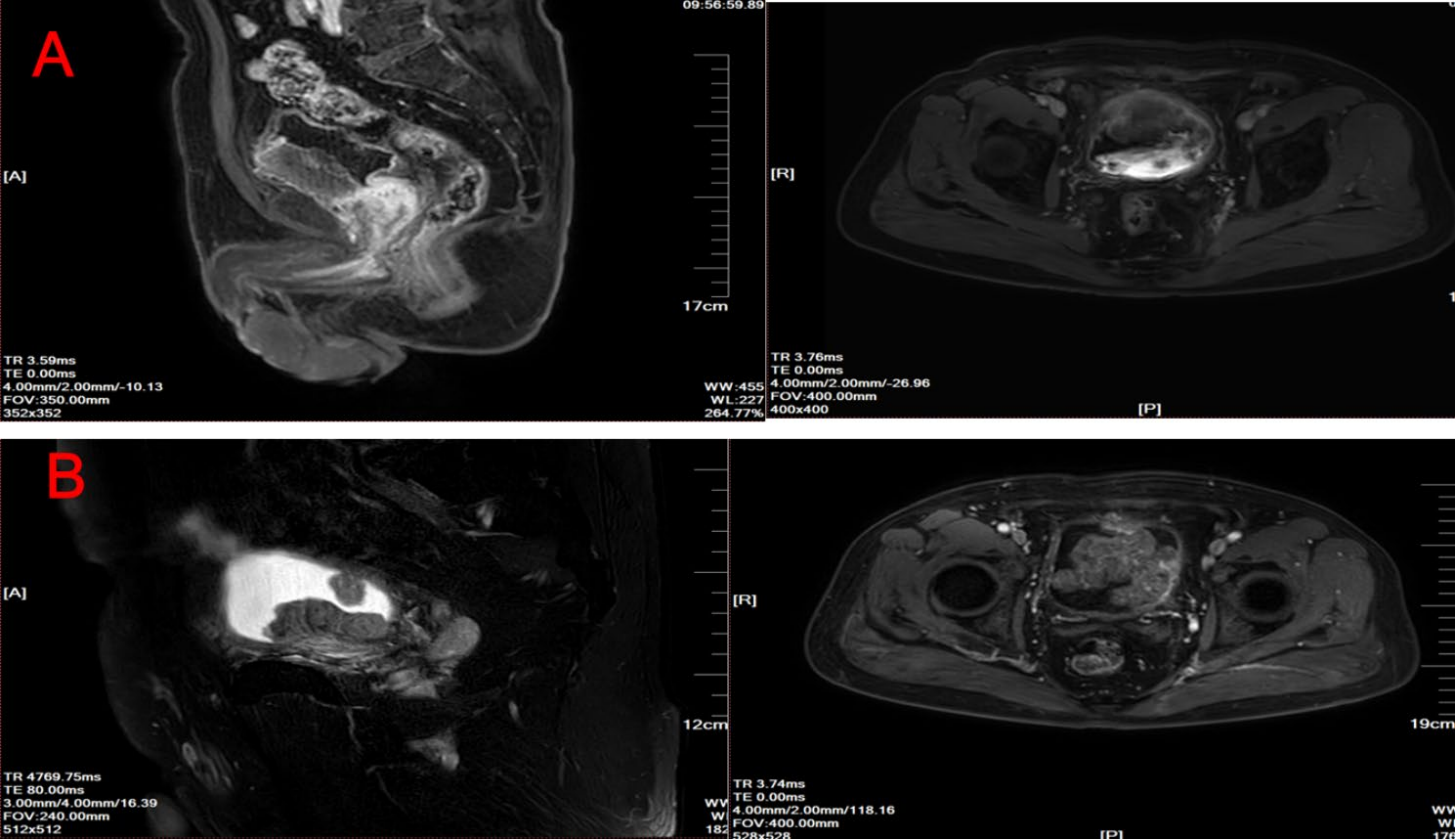
Magnetic resonance imaging of bladder cancer patient. **A)** Pelvic MRI showing signal abnormalities, the largest which had a size of 5.8*4 cm in the bladder, nodular signal shadow in prostate central zone (sagittal and coronal) in January 2021. **B)** Pelvic MRI showing irregular and abnormal signal shadow with a size of 8.4*6 cm in the bladder, an abnormal signal in the central gland of the prostate (sagittal and coronal) in May, 2021

**Fig. 4:**
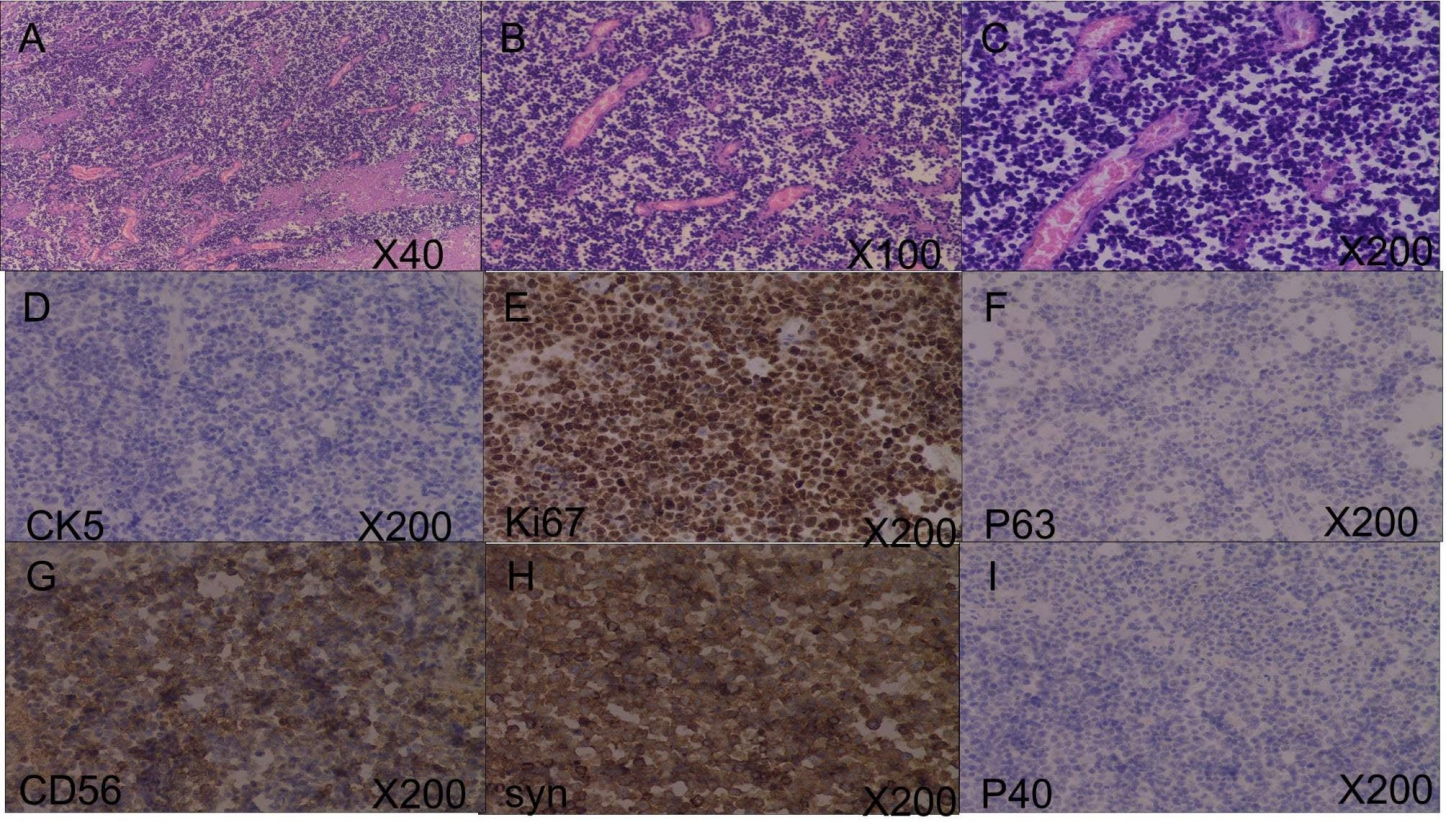
Pathological findings showing that the tumor in the bladder was small cell carcinoma and exhibited full-thickness invasion and high-grade urothelial cancer involving the prostate. Hematoxylin and eosin (H&E)-stained sections at: **(A)** × 40; **(B)** × 100; **(C)** × 200 magnification; **(D)** CK5 staining, ×200. **(E)** Ki67 staining, ×200. **(F)** P63 staining, ×200. **(G)** CD56 staining, ×200. **(H)** syn staining, ×200. **(I)** P40 staining, ×200

**Fig. 5:**
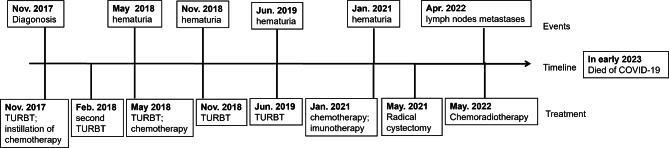
Sequence of events since diagnosis and a summary of the administered treatments

In January 2021, the patient presented once again with the manifestation of hematuria. Subsequent pelvic MRI examination revealed the presence of signal abnormalities, with the largest abnormality measuring approximately 5.8*4 cm, localized within the bladder. We also observed nodular signal shadow in the prostate central zone (Fig. 3A). Based on this finding, we hypothesized that the tumor would metastasize to the prostate. Subsequently, the was put on chemotherapy “gemcitabine 1.6 g (1 g/m^2^) D1, D8 + cisplatin 40 mg (25 mg/m^2^) D1-3” on 26th January and 13th March, 2021, respectively. He was also subjected to immunotherapy “tislelizumab 200 mg” on 2nd February, 23th February and 16th March, 2021. However, subsequent pelvic MRI, performed on 5th May, 2021, revealed irregular and abnormal signal shadow measuring 8.4*6 cm in his bladder, as well as an abnormal signal in the central gland of his prostate (Fig. 3B). Moreover, hematuria of this patient was difficult to control. Radical cystectomy, comprising total cystectomy, pelvic lymph node resection and cutaneous ureterostomy, was performed on 13th May, 2021. The pathological results confirmed that the tumor was SCC, and exhibited full-thickness invasion and high-grade urothelial cancer involving the prostate (Fig. 4). This was different from previous pathological findings. Immunohistochemical analysis of tumor tissues revealed the following results: CK8/18 (few +), CK (few +), CK5/6(-), P63(-), P40(-), Uroplakin III(+), GATA(-), CK7(few +), CK20(few +), CD56(+), Syn(+), CgA(few +),CD20(-), CD3(-), CD30(-), P53 mutant, and Ki-67(80%). Consequently, we recommended adjuvant chemotherapy or immunotherapy due to the presence of SCC. However, the patient could not adhere to the recommended treatment schedule due to economic reasons. In October, 2021, the patient was administered with antimicrobials due to urinary tract infection. Clinical examination revealed no abnormal findings. However, neck and mediastinum lymph nodes metastases were detected 11 months after radical cystectomy and a pathological diagnosis of SCC was made. Chemoradiotherapy was subsequently prescribed. Unfortunately, this patient died of COVID-19 in early 2023. The sequence of events since diagnosis have been summarized as shown in Fig. 5.

## Discussion and conclusions

Bladder cancer is a disease characterized by high recurrence [15]. In the majority of cases, the pathology observed in recurrent patients tends to align with the primary pathology. Previous studies have shown that collision tumor is not common among clinical patients, and SCC in bladder is a rare and aggressive malignant tumour [16]. SCC, complicated with urothelial carcinoma, has been previously reported [16]. In the present study, we report a case of a patient with high-grade papillary carcinoma that transformed to collision tumor with SCC. Since the mechanism underlying this transformation has not been previously explained, we postulate that the collision tumor was caused by a multiple of factors. Firstly, a common clonality maybe exist between SCC and urothelial carcinoma. A previous study detected TERT promoter mutations in urothelial carcinoma and SCC cases, which resulted in a common clonality [17]. Oncogenic factors, such as P53, VEGF, and EGFR, are known to play important roles in the signal transduction pathways of collision tumors [18]. Secondly, polyclonal cells are produced in the early stage of tumorigenesis. The close proximity of these cells may give rise to the development of two distinct and independent tumor types [19]. Furthermore, it is important to note that interrupted chemotherapy and surgical interventions in patients can directly contribute to the occurrence of mutations in tumors. Certain cancer therapies can induce DNA damage, leading to mutations in both malignant and non-malignant cells [20]. Inadequate treatment or treatment discontinuation which causes drug resistance or occurrence of mutations in tumor heterogeneity may be the underlying mechanisms driving the development of collision tumors. However, further research is required to explore the exact mechanisms. The likelihood of SCC originating from the prostate gland and infiltrating the bladder has been ruled out because no SCC cells were detected within the prostate gland. Moreover, there have been no reported cases of a single tumor type transforming into a collision tumor. Therefore, the mechanism underlying this transformation still remains unclear.

Previous studies have shown that bladder cancer patients have a five-year overall survival rate of 77% [21], while repeated TURBT has been recommended for high-risk NMIBC patients [22]. Another study reported that BCG had poor treatment efficacy in NMIBC patients at a higher risk of recurrence [23]. On the other hand, radical cystectomy has been advised for bladder patients at the highest risk [24]. Moreover, early-stage bladder cancer patients who received urothelial carcinoma radical nephroureterectomy exhibited better prognosis [25]. Although we recommended (on multiple occasions) radical cystectomy, the patient opted for interrupted chemotherapy and surgery due to concerns about potential adverse effects on their overall quality of life. This decision may have contributed to the development of the collision tumor observed in this case. Therefore, radical cystectomy needs to be performed early for NMIBC patients with a high risk of recurrence. Moreover, standardized and persistent treatment was necessary for NMIBC.

Previous studies have shown that adjuvant chemotherapy is beneficial for MIBC patients [26], while a combination of chemotherapy and radical cystectomy is recommended for patients with SCC bladder cancer [27]. Since the therapy is performed depending on the type of pathology, there is need to clearly define the pathology, even for patients who had relapsed, since collision tumor or other pathological tumor may be present. Treatments based on immune checkpoint inhibitors have achieved promising results in patients with SCC of urinary bladder [28]. It is a pity that this patient refused to stay on schedule for adjuvant chemotherapy or immunotherapy. Nevertheless, it is evident that standardized and persistent treatment can improve the survival rate in patients.

In conclusion, standardized treatment is essentially, especially for urinary system symptoms. Patients with such conditions should be administered with latest treatments, and strategies to improve adherence to treatment should be developed. It is important to emphasize that nonadherence to recommended clinical guidelines by patients may also affect disease progression. Notably, recurrent pathology in patients with bladder cancer should not be assumed to be necessarily the same tissue type as the primary pathology. Pathological examination should be conducted to confirm the pathological diagnosis and guide the selection of targeted clinical treatment. Although the probability of collision tumor is low, clinicians should be vigilant about the disease.

Standardized and persistent treatment is of utmost importance in the management of NMIBC. A clear and well-defined pathological analysis should be conducted to guide the selection of appropriate therapies tailored to the specific pathology, even in cases of disease recurrence. This approach is crucial as it takes into account the potential presence of collision or other pathological tumors. We recommend early radical cystectomy for NMIBC patients at a high risk of recurrence. However, this conclusion needs to be validated in a large number of patients.

## Data Availability

All datasets generated in this study are included in the manuscript.

## List of Abbreviations

BCG: Bacillus Calmette Guerin
MIBC: Muscular invasive bladder cancer
MRI: Magnetic resonance imaging
NMIBC: Non-muscle invasive bladder cancer
SCC: Small cell carcinoma
TURBT: Transurethral resection of bladder tumor
UICC: Union for International Cancer Control
